# Supplementary material for: Automated screening of sickle cells using a smartphone-based microscope and deep learning
Source: NPJ Digit Med. 2020 May 22;3:76. doi: 10.1038/s41746-020-0282-y (PMC7244537; doi:10.1038/s41746-020-0282-y)
Supplement: Supplementary file 1 — Supplementary Information [file 41746_2020_282_MOESM1_ESM.pdf]

Supplementary materials

Automated screening of sickle cells using a smartphone-based microscope and deep learning

Kevin de Haan[†,1,2,3], Hatice Ceylan Koydemir[†,1,2,3], Yair Rivenson[1,2,3,*], Derek Tseng[1,2,3], Elizabeth Van Dyne[4], Lissette Bakic[5], Doruk Karinca[6], Kyle Liang[6], Megha Ilango[6], Esin Gumustekin[7], Aydogan Ozcan[1,2,3,8,*]

[1]Electrical and Computer Engineering Department, University of California, Los Angeles, CA, 90095, USA.

[2]Bioengineering Department, University of California, Los Angeles, CA, 90095, USA.

[3]California NanoSystems Institute (CNSI), University of California, Los Angeles, CA, 90095, USA.

[4] Department of Pediatrics, Division of Hematology-Oncology, David Geffen School of Medicine, University of California, Los Angeles, CA, 90095, USA.

[5]Department of Pathology and Laboratory Medicine, David Geffen School of Medicine, University of California, Los Angeles, Los Angeles, CA, USA

[6]Department of Computer Science, University of California, Los Angeles, CA, 90095, USA.

[7]Department of Neuroscience, University of California, Los Angeles, CA, 90095, USA.

[8]Department of Surgery, David Geffen School of Medicine, University of California, Los Angeles, CA, 90095, USA.

† Equally contributing authors

* Corresponding authors: rivensonyair@ucla.edu ; ozcan@ucla.edu

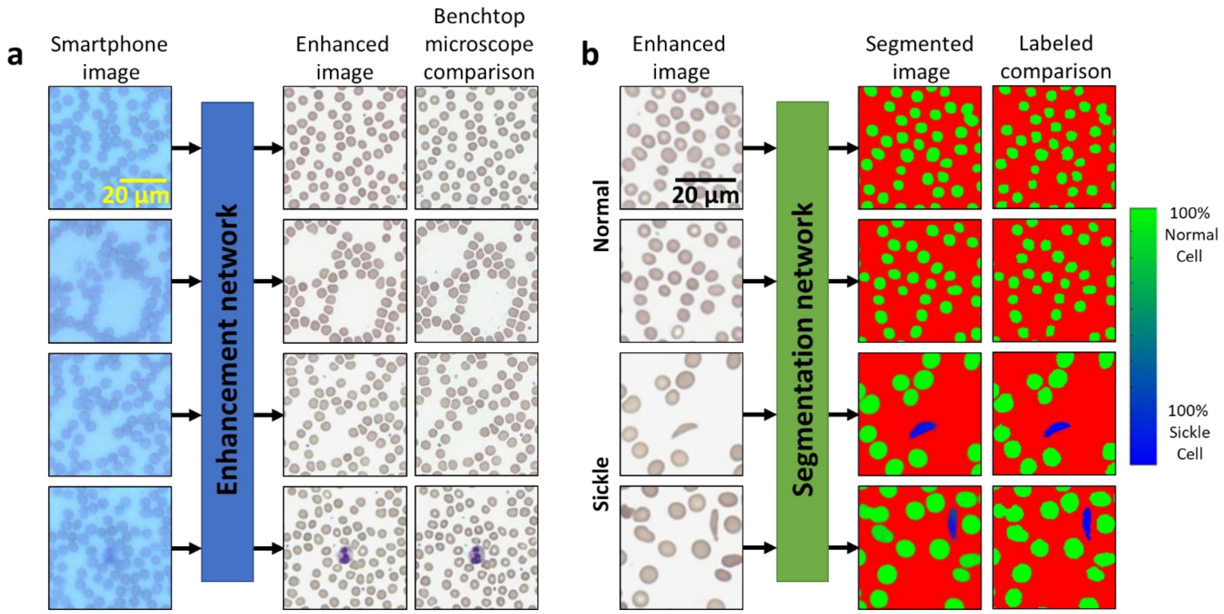

Supplementary Figure 1. *Direct comparison between the network output and the ground truth for the two networks. a) Demonstration of the image enhancement network, where the smartphone image is the network input, the enhanced image is the network output, and the benchtop microscope (NA=0.75) is the ground truth image label. b) Demonstration of the segmentation network, where the enhanced image is the network input, a segmented probability map is the network output, and the probability mask based on the expert's label is the ground truth.*

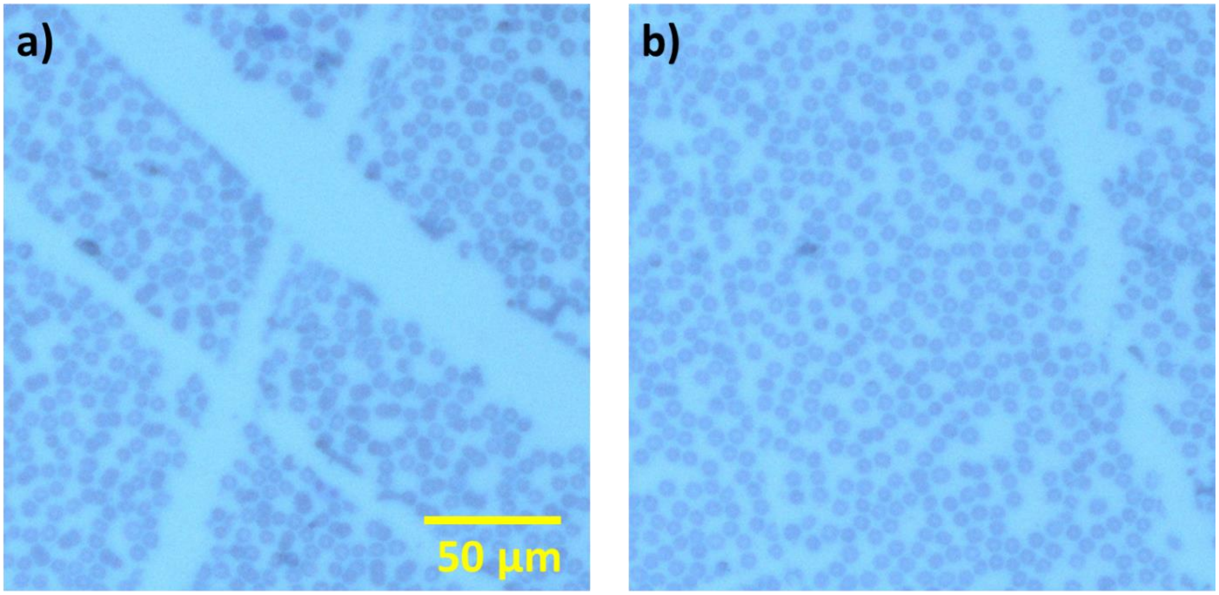

Supplementary Figure 2. *Examples of two areas of a normal blood smear which has been scraped. Several of the cells in each field of view have become sickle-shaped due to being cut. Such slides were excluded in our training and testing.*

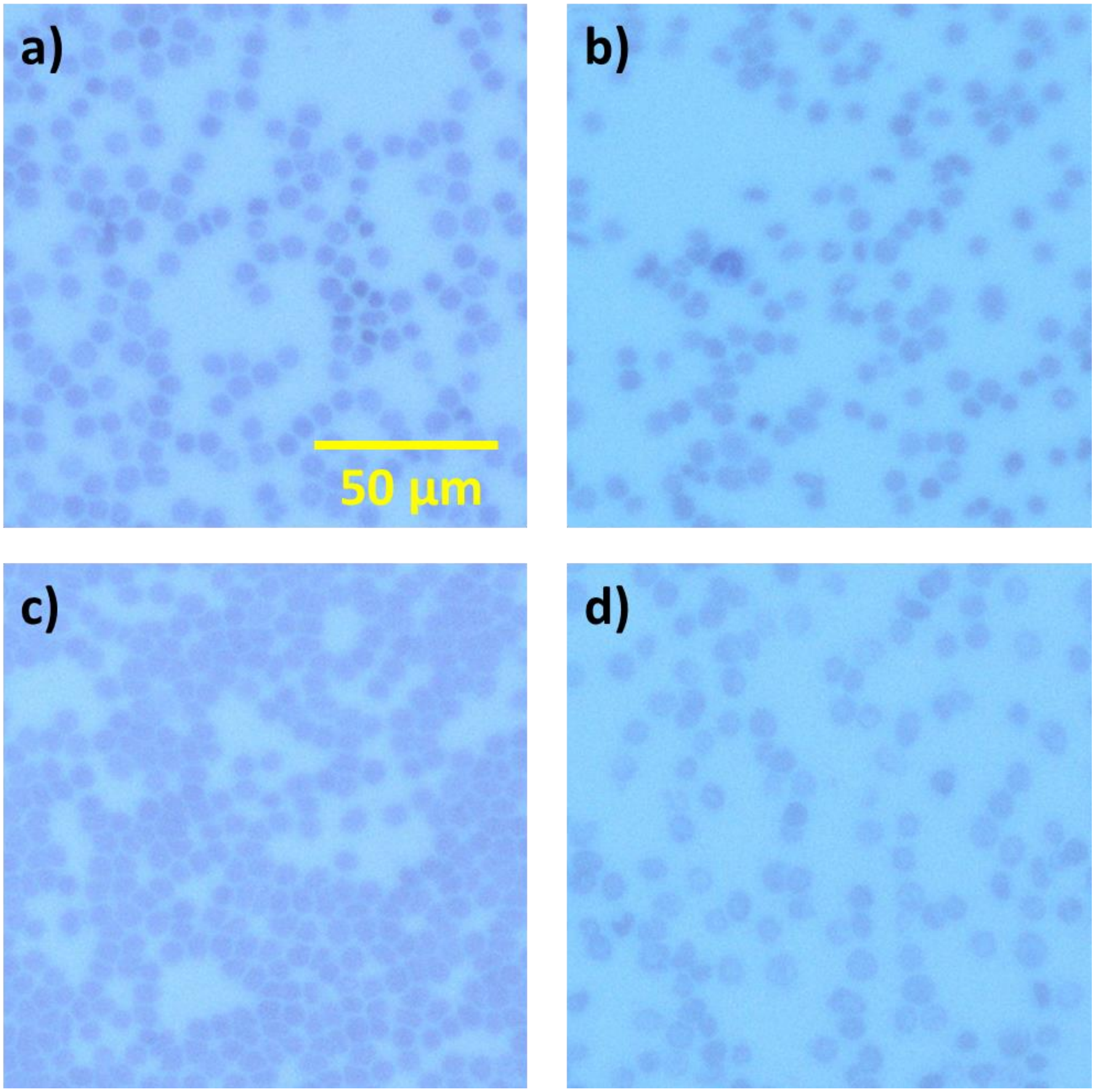

Supplementary Figure 3. *Demonstration of the aberrations and various differences among smartphone microscope images. Aberrations include blur due to the device's inherent challenge to accurately focus on the sample, as well as blur in certain areas of the image due to the usage of cost-effective optical components, tilt and defocus aberrations due to optical misalignments, spectral aberrations between the fields of view as well as between the cells within each field of view, and pixel saturation in the centers of some of the cells (e.g., the cells in panel (d)).*

Supplementary Table 1. *Percentage of sickle cells measured by our system from 32 SCD blood smears and 64 healthy blood smears. A slide was classified as positive for SCD if the average sickle cell percentage is over 0.5% (which is determined at the validation phase, before the blind testing). Slides which were either false positives or false negatives are marked in red.*

Slide Number	Sickle %	Slide Number	Sickle %	Slide Number	Sickle %
Sickle 1	0.66	Healthy 1	0.15	Healthy 33	0.09
Sickle 2	2.35	Healthy 2	0.27	Healthy 34	0.15
Sickle 3	0.54	Healthy 3	0.32	Healthy 35	0.28
Sickle 4	1.31	Healthy 4	0.13	Healthy 36	0.15
Sickle 5	0.78	Healthy 5	0.21	Healthy 37	0.33
Sickle 6	4.38	Healthy 6	0.10	Healthy 38	0.17
Sickle 7	1.99	Healthy 7	0.10	Healthy 39	0.01
Sickle 8	0.46	Healthy 8	0.33	Healthy 40	0.40
Sickle 9	0.91	Healthy 9	0.08	Healthy 41	0.29
Sickle 10	6.51	Healthy 10	0.04	Healthy 42	0.32
Sickle 11	9.73	Healthy 11	0.00	Healthy 43	0.07
Sickle 12	4.52	Healthy 12	0.01	Healthy 44	0.06
Sickle 13	10.46	Healthy 13	0.19	Healthy 45	0.02
Sickle 14	5.04	Healthy 14	0.10	Healthy 46	0.11
Sickle 15	9.89	Healthy 15	0.08	Healthy 47	0.00
Sickle 16	4.16	Healthy 16	0.24	Healthy 48	0.17
Sickle 17	1.42	Healthy 17	0.07	Healthy 49	0.08
Sickle 18	1.32	Healthy 18	0.64	Healthy 50	0.03
Sickle 19	0.56	Healthy 19	0.07	Healthy 51	0.04
Sickle 20	0.74	Healthy 20	0.23	Healthy 52	0.17
Sickle 21	8.65	Healthy 21	0.06	Healthy 53	0.08
Sickle 22	0.58	Healthy 22	0.02	Healthy 54	0.24
Sickle 23	2.88	Healthy 23	0.21	Healthy 55	0.10
Sickle 24	2.15	Healthy 24	0.19	Healthy 56	0.31
Sickle 25	0.51	Healthy 25	0.36	Healthy 57	0.09
Sickle 26	4.21	Healthy 26	0.25	Healthy 58	0.06
Sickle 27	4.82	Healthy 27	0.34	Healthy 59	0.02
Sickle 28	1.03	Healthy 28	0.34	Healthy 60	0.05
Sickle 29	3.75	Healthy 29	0.12	Healthy 61	0.00
Sickle 30	1.28	Healthy 30	0.37	Healthy 62	0.04
Sickle 31	2.81	Healthy 31	0.03	Healthy 63	0.00
Sickle 32	2.14	Healthy 32	0.02	Healthy 64	0.11
